# *C* ^1^-Almost Periodic Solutions of BAM Neural Networks with Time-Varying Delays on Time Scales

**DOI:** 10.1155/2015/727329

**Published:** 2015-01-19

**Authors:** Yongkun Li, Lili Zhao, Li Yang

**Affiliations:** Department of Mathematics, Yunnan University, Kunming, Yunnan 650091, China

## Abstract

On a new type of almost periodic time scales, a class of BAM neural networks is considered. By employing a fixed point theorem and differential inequality techniques, some sufficient conditions ensuring the existence and global exponential stability of *C* ^1^-almost periodic solutions for this class of networks with time-varying delays are established. Two examples are given to show the effectiveness of the proposed method and results.

## 1. Introduction

It is well known that bidirectional associative memory (BAM) neural networks have been extensively applied within various engineering and scientific fields such as pattern recognition, signal and image processing, artificial intelligence, and combinatorial optimization [1–3]. Since all these applications closely relate to the dynamics, the dynamical behaviors of BAM neural networks have been widely investigated. There have been extensive results on the problem of the existence and stability of equilibrium points, periodic solutions, and antiperiodic solutions of BAM neural networks in the literature. We refer the reader to [4–16] and the references cited therein. Moreover, it is known that the existence and stability of almost periodic solutions play a key role in characterizing the behavior of dynamical systems (see [17–26]) and the *C* ^1^-almost periodic function is an important subclass of almost periodic functions. However, to the best of our knowledge, few authors have studied problems of *C* ^1^-almost periodic solutions of BAM neural networks.

On the other hand, the theory of calculus on time scales (see [27, 28] and references cited therein) was initiated by Hilger in his Ph.D. thesis in 1988 in order to unify continuous and discrete analyses, and it helps avoid proving twice results, once for differential equations and once for difference equations. Therefore, it is significant to study neural networks on time scales (see [5, 29, 30]). In fact, both continuous-time and discrete-time BAM-type neural networks have equal importance in various applications. But it is troublesome to study the existence and stability of almost periodic and *C* ^1^-almost periodic solutions for continuous and discrete systems, respectively. Motivated by the above, our purpose of this paper is to study the existence and stability of *C* ^1^-almost periodic solutions for the following BAM neural networks on time scales:

(1) xiΔt=−aitxit+∑j=1mpjitfjyjt−γjit+Iit,00000000000000000000000000000 t∈T, i=1,2,…,n,yjΔt=−bjtyjt+∑i=1nqijtgixit−ρijt+Jjt,0000000000000000000000000000 t∈T, j=1,2,…,m,

where *T* is an almost periodic time scale which will be defined in the next section; *x*_*i*_(*t*) and *y*_*j*_(*t*) correspond to the activation of the *i*th neurons and the *j*th neurons at the time *t*, respectively; *a*_*i*_(*t*), *b*_*j*_(*t*) are positive functions and they denote the rates with which the cells *i* and *j* reset their potential to the resting state when isolated from the other cells and inputs at time *t*; *p*_*ji*_(*t*) and *q*_*ij*_(*t*) are the connection weights at time *t*; *γ*_*ji*_(*t*), *ρ*_*ij*_(*t*) are nonnegative, which correspond to the finite speed of the axonal signal transmission at time *t*; *I*_*i*_(*t*), *J*_*j*_(*t*) denote the external inputs at time *t*; and *f*_*j*_ and *g*_*i*_ are the activation functions of signal transmission. For each interval *J* of *R*, we denote *J*_*T*_ = *J*∩*T*.

Throughout this paper, we assume the following:

(*H*_1_)
*f*_*j*_, *g*_*i*_ ∈ *C*(*R*, *R*) and there exist positive constants *α*_*j*_, *β*_*i*_ such that
  (2) fju−fjvαju−v,giu−giv≤βiu−v,
 where |*u* | , |*v* | ∈ *R*, *i* = 1,2,…, *n*, *j* = 1,2,…, *m*;
(*H*_2_)
*a*_*i*_(*t*) > 0, *b*_*j*_(*t*) > 0, *γ*_*ji*_(*t*) ≥ 0, *ρ*_*ij*_(*t*) ≥ 0, *p*_*ji*_(*t*), *q*_*ij*_(*t*), *I*_*i*_(*t*), *J*_*j*_(*t*) are bounded almost periodic functions on *T*, *i* = 1,2,…, *n*, *j* = 1,2,…, *m*.

System (1) is supplemented with the initial values given by

(3) WWWWxis=φis,WWWWyjs=φn+js,i=1,2,…,n, j=1,2,…,m,

where *φ*_*k*_(·) denotes a real-valued bounded rd-continuous function defined on [−*v*, 0]_*T*_, and

(4) γimax⁡1≤j≤m sup⁡t∈T γjit,γ=max⁡1≤i≤n γi,ρj=max⁡1≤i≤n sup⁡t∈T ρijt,ρ=max⁡1≤j≤m ρj,v=max⁡γ,ρ.

## 2. Preliminaries

In this section, we will first recall some basic definitions and lemmas which are used in what follows.

Let *T* be a nonempty closed subset (time scale) of *R*. The forward and backward jump operators *σ*, *ρ* : *T* → *T* and the graininess *μ* : *T* → *R* ^+^ are defined, respectively, by

(5) σtinf⁡⁡s∈T:s>t,ρt=sup⁡s∈T:s<t,μt=σt−t.

A point *t* ∈ *T* is called left-dense if *t* > inf⁡*T* and *ρ*(*t*) = *t*, left-scattered if *ρ*(*t*) < *t*, right-dense if *t* < sup⁡*T* and *σ*(*t*) = *t*, and right-scattered if *σ*(*t*) > *t*. If *T* has a left-scattered maximum *m*, then *T* ^*k*^ = *T*∖{*m*}; otherwise, *T* ^*k*^ = *T*. If *T* has a right-scattered minimum *m*, then *T*_*k*_ = *T*∖{*m*}; otherwise, *T*_*k*_ = *T*.

A function *f* : *T* → *R* is right-dense continuous provided it is continuous at right-dense point in *T* and its left-side limits exist at left-dense points in *T*. If *f* is continuous at each right-dense point and each left-dense point, then *f* is said to be continuous function on *T*.

For *y* : *T* → *R* and *t* ∈ *T* ^*k*^, we define the delta derivative of *y*(*t*), *y* ^Δ^(*t*), to be the number (if it exists) with the property that for a given *ε* > 0 there exists a neighborhood *U* of *t* such that

(6) $$\begin{array}{c} \left|\left[y\left(\sigma \left(t\right)\right)-y\left(s\right)\right]-y^{\Delta }\left(t\right)\left[\sigma \left(t\right)-s\right]\right|<\varepsilon \left|\sigma \left(t\right)-s\right| \end{array}$$

for all *s* ∈ *U*.

If *y* is continuous, then *y* is right-dense continuous, and if *y* is delta differentiable at *t*, then *y* is continuous at *t*.

Let *y* be right-dense continuous. If *Y* ^Δ^(*t*) = *y*(*t*), then we define the delta integral by

(7) $$\begin{array}{c} \overset{t}{\underset{a}{\int }}y\left(s\right)\Delta s=Y\left(t\right)-Y\left(a\right). \end{array}$$

A function *r* : *T* → *R* is called regressive if

(8) $$\begin{array}{c} 1+\mu \left(t\right)r\left(t\right)\neq 0 \end{array}$$

for all *t* ∈ *T* ^*k*^. The set of all regressive and rd-continuous functions *r* : *T* → *R* will be denoted by *R* = *R*(*T*) = *R*(*T*, *R*). We define the set *R* ^+^ = *R* ^+^(*T*, *R*) = {*r* ∈ *R* : 1 + *μ*(*t*)*r*(*t*) > 0, ∀*t* ∈ *T*}.

If *r* is a regressive function, then the generalized exponential function *e*_*r*_ is defined by

(9) $$\begin{array}{c} e_{r}\left(t,s\right)=\exp\left\{\overset{t}{\underset{s}{\int }}\xi_{\mu \left(\tau \right)}\left(r\left(\tau \right)\right)\Delta \tau \right\},\;\text{for}\;\;s,t\in T, \end{array}$$

with the cylinder transformation

(10) $$\begin{array}{c} \xi_{h}\left(z\right)=\left\{\begin{array}{cc} \frac{\text{Log}\left(1+hz\right)}{h} & \text{if}\;\;h\neq 0,\\ z & \text{if}\;\;h=0. \end{array}\right. \end{array}$$

Let *p*, *q* : *T* → *R* be two regressive functions; we define

(11) p⊕q≔p+q+μpq,⊖p≔−p1+μp,p⊖q≔p⊕⊖q.

Then the generalized exponential function has the following properties.

**Lemma 1 (see [31]).**

Assume that *p*, *q* : *T* → *R* are two regressive functions; then,

- *e*_0_(*t*, *s*) ≡ 1 and *e*_*p*_(*t*, *t*) ≡ 1;
- *e*_*p*_(*σ*(*t*), *s*) = (1 + *μ*(*t*)*p*(*t*))*e*_*p*_(*t*, *s*);
- *e*_*p*_(*t*, *σ*(*s*)) = *e*_*p*_(*t*, *s*)/(1 + *μ*(*s*)*p*(*s*));
- 1/*e*_*p*_(*t*, *s*) = *e*_⊖*p*_(*t*, *s*);
- (*e*_⊖*p*_(*t*, *s*))^Δ^ = (⊖*p*)(*t*)*e*_⊖*p*_(*t*, *s*).

In this section, *E* ^*n*^ denotes *R* ^*n*^ or *C* ^*n*^, *D* denotes an open set in *E* ^*n*^ or *D* = *E* ^*n*^, and *S* denotes an arbitrary compact subset of *D*.

**Definition 2 .**

A time scale *T* is called an almost periodic time scale if

(12) $$\begin{array}{c} \Pi ≔\left\{\tau \in R:T_{\tau }\neq \phi \right\}\neq \left\{0\right\} \end{array}$$

satisfies that, for any *τ*_1_, *τ*_2_ ∈ Π, one has *τ*_1_ ± *τ*_2_ ∈ Π, where *T*_*τ*_ = *T*∩{*T* − *τ*}.

**Definition 3 .**

Let *T* be an almost periodic time scale. For any *t* ∈ *T*, *τ* ∈ Π, we define

(13) $$\begin{array}{c} t\tilde{+}\tau =\left\{\begin{array}{cc} t+\tau & \text{if}\;\;t\in \tilde{T},\\ t & \text{if}\;\;t\notin \tilde{T}, \end{array}\right. \end{array}$$

where $\tilde{T}={⋂}_{\tau \in \Pi }T_{\tau }$.

Obviously, if *T* is an almost periodic time scale, then inf⁡*T* = −*∞* and sup⁡*T* = +*∞*. If there exists a *τ* ∈ Π such that *T*_*τ*_ = *T*, then Definition 2 is equivalent to Definition 3.7 in [31]; otherwise, Definition 2 is more general than Definition 3.7 in [31].

**Definition 4 .**

Let *T* be an almost periodic time scale. A function *f* ∈ *C*(*T* × *D*, *E* ^*n*^) is called an almost periodic function in *t* ∈ *T* uniformly for *x* ∈ *D* if the *ε*-translation set of *f*

(14) Eε,f,S =τ∈Π:ft+~τ,x−ft,x<ε,  ∀t,x∈T×S

is a relatively dense set in *T* for all *ε* > 0 and for each compact subset *S* of *D*; that is, for any given *ε* > 0 and each compact subset *S* of *D*, there exists a constant *l*(*ε*, *S*) > 0 such that each interval of length *l*(*ε*, *S*) contains a *τ*(*ε*, *S*) ∈ *E*{*ε*, *f*, *S*} such that

(15) $$\begin{array}{c} \left|f\left(t\tilde{+}\tau ,x\right)-f\left(t,x\right)\right|<\varepsilon ,\;\forall t\in T\times S. \end{array}$$

*τ* is called the *ε*-translation number of *f* and and *l*(*ε*, *S*) is called the inclusion length of *E*{*ε*, *f*, *S*}.

For convenience, we introduce some notations. Let *α* = {*α*_*n*_} and *β* = {*β*_*n*_} be two sequences. Then *β* ⊂ *α* means that *β* is a subsequence of *α*. We introduce the translation operator *T*, and *T*_*α*_ *f*(*t*, *x*) = *g*(*t*, *x*) means that $g\left(t,x\right)=\lim_{n\to +\infty }f\left(t\tilde{+}\alpha_{n},x\right)$. From Definitions 2 and 4, one can easily see that all the results obtained in [31] are still valid under the new concepts of almost periodic time scales and almost periodic functions on time scales. For example, similar to Theorems 3.13 and 3.14 in [31], we can obtain the following equivalent definition of uniformly almost periodic functions.

**Definition 5 .**

Let *f*(*t*, *x*) ∈ *C*(*T* × *D*, *E* ^*n*^), and if for any given sequence *α*′ ⊂ Π and each compact subset *S* of *D* there exists a subsequence *α* ⊂ *α*′ such that *T*_*α*_ *f*(*t*, *x*) exists uniformly on *T* × *S*, then *f*(*t*, *x*) is called an almost periodic function in *t* uniformly for *x* ∈ *D*.

**Definition 6 .**

A function *f* ∈ *C* ^1^(*T*, *R*) is said to be a *C* ^1^-almost periodic function, if *f*, *f* ^Δ^ are two almost periodic functions on *T*.

**Definition 7 (see [31]).**

Let *x* ∈ *R* ^*n*^, and let *A*(*t*) be an *n* × *n* rd-continuous matrix on *T*; the linear system

(16) $$\begin{array}{c} x^{\Delta }\left(t\right)=A\left(t\right)x\left(t\right),\;t\in T \end{array}$$

is said to admit an exponential dichotomy on *T* if there exist positive constants *k* and *α*, projection *P*, and the fundamental solution matrix *X*(*t*) of (15), satisfying

(17) XtPX−1σs0≤ke⊖αt,σs,hhhhhhhhhhhhhhhs,t∈T,  t≥σs,XtI−PX−1σs0≤ke⊖ασs,t,hhhhhhhhhhhhhhhhhhhhs,t∈T,  t≤σs,

where ‖·‖_0_ is a matrix norm on *T*.

Consider the following linear almost periodic system:

(18) $$\begin{array}{c} x^{\Delta }\left(t\right)=A\left(t\right)x\left(t\right)+f\left(t\right),\;t\in T, \end{array}$$

where *A*(*t*) is an almost periodic matrix function and *f*(*t*) is an almost periodic vector function.

**Lemma 8 (see [31]).**

If the linear system (15) admits exponential dichotomy, then system (16) has a unique almost periodic solution *x*(*t*):

(19) xt∫−∞tXtPX−1σsfsΔs−∫t+∞XtI−PX−1σsfsΔs,

where *X*(*t*) is the fundamental solution matrix of (15).

**Lemma 9 (see [24]).**

Let *c*_*i*_(*t*) be an almost periodic function on *T*, where *c*_*i*_(*t*) > 0, −*c*_*i*_(*t*) ∈ *R* ^+^, ∀*t* ∈ *T*, and

(20) $$\begin{array}{c} \underset{1\leq i\leq n}{\min}\left\{\underset{t\in T}{\inf}\;c_{i}\left(t\right)\right\}=\tilde{m}>0; \end{array}$$

then, the linear system

(21) $$\begin{array}{c} x^{\Delta }\left(t\right)=\mathrm{diag}\left(-c_{1}\left(t\right),-c_{2}\left(t\right),\ldots ,-c_{n}\left(t\right)\right)x\left(t\right) \end{array}$$

admits an exponential dichotomy on *T*.

**Lemma 10 (see [27]).**

Every rd-continuous function has an antiderivative. In particular, if *t*_0_ ∈ *T*, then *F* defined by

(22) $$\begin{array}{c} F\left(t\right)=\overset{t}{\underset{t_{0}}{\int }}f\left(s\right)\Delta s,\;t\in T \end{array}$$

is an antiderivative of *f*.

**Lemma 11 (see [27]).**

If *p* ∈ *R* and *a*, *b*, *c* ∈ *T*, then

(23) epc,·Δ−pepc,·σ,∫abptepc,σtΔt=epc,a−epc,b.

By Lemmas 10 and 11, it is easy to get the following lemma.

**Lemma 12 .**

Suppose that *f*(*t*) is an *rd*-continuous function and *c*(*t*) is a positive *rd*-continuous function which satisfies that −*c*(*t*) ∈ *R* ^+^. Let

(24) $$\begin{array}{c} g\left(t\right)=\overset{t}{\underset{t_{0}}{\int }}e_{-c}\left(t,\sigma \left(s\right)\right)f\left(s\right)\Delta s, \end{array}$$

where *t*_0_ ∈ *T*; then,

(25) $$\begin{array}{c} g^{\Delta }\left(t\right)=f\left(t\right)+\overset{t}{\underset{t_{0}}{\int }}-c\left(t\right)e_{-c}\left(t,\sigma \left(s\right)\right)f\left(s\right)\Delta s. \end{array}$$

**Lemma 13 (see [31]).**

If *g* is a real-valued almost periodic function on *T* and *f* : *R* → *R* is a Lipschitz function, then *t* → *f*(*g*(*t*)) is an almost periodic function on *T*.

**Lemma 14 (see [31]).**

If *f*, *g* : *T* → *R* are almost periodic functions, then the following hold:

1. *f* + *g* is almost periodic function;
2. *fg* is almost periodic function.

## 3. Existence of *C* ^1^-Almost Periodic Solutions

First, for convenience, we introduce some notations. We will use *x* = (*x*_1_, *x*_2_,…, *x*_*n*+*m*_)^*T*^ ∈ *R* ^*n*+*m*^ to denote a column vector, in which the symbol *T* denotes the transpose of vector. We let |*x*| denote the absolute-value vector given by |*x* | = (|*x*_1_ | , |*x*_2_ | ,…, |*x*_*n*+*m*_ | )^*T*^ and define ‖*x*‖ = max⁡_1≤*i*≤*n*+*m*_|*x*_*i*_|.

Let *AP*(*T*) = {*c*(*t*) : *c*(*t*) be a bounded real-valued, almost periodic function on *T*}, *AP* ^1^(*T*) = {*c*(*t*) : *c*(*t*), *c* ^Δ^(*t*) ∈ *AP*(*T*)}, and

(26) B=φ=φ1t,φ2t,…,φnt,φn+1t,…,φn+mtT:0000φit∈AP1T, i=1,2,…,n+mφ1t,φ2t,…,φnt,φn+1t,…,φn+mtT.

For ∀*φ* ∈ *B*, if we define induced modulus

(27) $$\begin{array}{c} {\left\|\varphi \right\|}_{B}=\underset{t\in T}{\sup}{\left\|\varphi \right\|}_{1}=\max\left\{{\left\|\varphi \right\|}_{0},{\left\|\varphi^{\Delta }\right\|}_{0}\right\}, \end{array}$$

where

(28) WWφt1=max⁡φt0,φΔt0,WWφt0=max⁡1≤i≤n+mφt,WWφ0=sup⁡t∈Tφt0,WφΔt =φ1Δt,…,φnΔt,φn+1Δt,…,φn+mΔtT,

then *B* is a Banach space.

**Theorem 15 .**

Assume that (*H*_1_), (*H*_2_), and the following hold:

(*H*_3_)
−*a*_*i*_, −*b*_*j*_ ∈ *R* ^+^, *t* − *γ*_*ji*_(*t*), *t* − *ρ*_*ij*_(*t*) ∈ *T*, ∀*t* ∈ *T*, *i* = 1,2,…, *n*, *j* = 1,2,…, *m*;
(*H*_4_)
there exists a constant *r*_0_ such that
  (29) max⁡1≤i≤n,1≤j≤mai¯+ai_ai_ηi,bj¯+bj_bj_ηj¯+max⁡L1,L2≤r0,00000000000000000000000000  0<Πi<ai_ai¯+ai_<ai_,00000000000000000000000000 0<Πj¯<bj_bj¯+bj_<bj_,000000000000000000 i=1,2,…,n, j=1,2,…,m,

where

(30) ηi∑j=1mpji¯fj0+αjr0,ηj¯=∑i=1nqij¯gi0+βir0,Πi=∑j=1mpji¯αj,Πj¯=∑i=1nqij¯βi,L1=max⁡1≤i≤nai¯+ai_ai_Ii¯,L2=max⁡1≤j≤mbj¯+bj_bj_Jj¯,ai_=inf⁡t∈T ait,bj_=inf⁡t∈T bjt,ai¯=sup⁡t∈T ait,bj¯=sup⁡t∈T bjt,Ii¯=sup⁡t∈TIit,Jj¯=sup⁡t∈TJjt,pji¯=sup⁡t∈Tpjit,qij¯=sup⁡t∈Tqijt;

then, system (1) has a unique *C* ^1^-almost periodic solution in the region

(31) $$\begin{array}{c} E=\left\{\varphi \in B:{\left\|\varphi \right\|}_{B}\leq r_{0}\right\}. \end{array}$$

**Proof**

For any given *φ* ∈ *B*, we consider the following almost periodic differential equation:

(32) xiΔt−aitxit+∑j=1mpjitfjφn+jt−γjit+Iit, i=1,2,…,n,yjΔt−bjtyjt+∑i=1nqijtgiφit−ρijt+Jjt, j=1,2,…,m.

Since min⁡_1≤*i*≤*n*,1≤*j*≤*m*_{inf⁡_*t*∈*T*_ *a*_*i*_(*t*), inf⁡_*t*∈*T*_ *b*_*j*_(*t*)} > 0, it follows from Lemma 10 that the linear system

(33) xiΔt−aitxit, i=1,2,…,n,yjΔt−bjtyjt, j=1,2,…,m,

admits an exponential dichotomy on *T*. Thus, by Lemma 9, we know that system (32) has exactly one almost periodic solution:

(34) xφitW=∫−∞te−ait,σsWWWW·∑j=1mpjisfjφn+js−γjis+IisΔs,yφn+jtW=∫−∞te−bjt,σsWWWW·∑i=1nqijsgiφis−ρijs+JjsΔs.

By Lemmas 13 and 14, we have that

(35) xφiΔtW=∑j=1mpjitfjφn+jt−γjit+IitWW−ait∫−∞te−ait,σsWWWWWWW·∑j=1mpjisfjWWWWWWWWWW·φn+js−γjis+Iis∑j=1mpjiΔs,WWWWWWWWWWWWWWW00WWi=1,2,…,n,yφn+jΔt=∑i=1nqijtgiφit−ρijt+JjtW−bjt∫−∞te−bjt,σsWWWWWW·∑i=1nqijsgiφis−ρijs+JjsΔs,WWWWWWWWW0WWWWWWWWj=1,2,…,m,

are almost periodic functions on *T*; that is, (34) is not only an almost periodic solution of system (32), but also a *C* ^1^-almost periodic solution of system (32). First, we define a nonlinear operator on *B* by

(36) ΦφtW=xφ1t,xφ2t,…,xφnt,yφn+1t,…,yφn+mtT,WWWWWWWWWWWWWWWW0WWWW∀φ∈B.

Next, we check that Φ(*E*) ⊂ *E*. For any given *φ* ∈ *E*, it suffices to prove that ‖Φ(*φ*)‖_*B*_ ≤ *r*_0_. By conditions (*H*_1_)–(*H*_4_), we have

(37) sup⁡t∈Txφit=sup⁡t∈T∑j=1mpji∑j=1mpjisfjφn+js−γjis+Iis∫−∞te−ait,σsWWWWWW·∑j=1mpjisfjφn+js−γjisWWWWWWWWW+Iis∑j=1mpjiΔs≤sup⁡t∈T∑j=1mpji¯fjφn+js−γjis∫−∞te−ai_t,σsWWWWW·∑j=1mpji¯fjφn+js−γjisΔs+Ii¯ai_≤sup⁡t∈T+αjφn+js−γjis∑j=1mpji¯Δs∫−∞te−ai_t,σsWWWWW·∑j=1mpji¯fj0WWWWWWWWW+αjφn+js−γjis∑j=1mpji¯ΔsM+Ii¯ai_≤sup⁡t∈T∑j=1mpji¯fj0+αjr0∫−∞te−ai_t,σsWWWWW·∑j=1mpji¯fj0+αjr0Δs+Ii¯ai_≤ηiai¯+ai¯+ai_ai_Ii¯≤ai¯+ai_ai_ηi+L1≤r0,WWWWWWWWWWWWWWWWWi=1,2,…,n,sup⁡t∈Tyφn+jt=sup⁡t∈T∑i=1nqijsgiφis−ρijs+Jjs∫−∞te−bjt,σsWWWWW·∑i=1nqijsgiφis−ρijs+JjsΔs≤sup⁡t∈T∑i=1nqij¯giφis−ρijs∫−∞te−bj_t,σsWWWWW·∑i=1nqij¯giφis−ρijsΔs+Jj¯bj_≤sup⁡t∈T∑i=1nqij¯gi0+βiφis−ρijs∫−∞te−bj_t,σsWWWWW·∑i=1nqij¯gi0+βiφis−ρijsΔsWW+Jj¯bj_≤sup⁡t∈T∑i=1nqij¯gi0+βir0∫−∞te−bj_t,σsWWWWW·∑i=1nqij¯gi0+βir0Δs+Jj¯bj_≤ηj¯bj_+bj¯+bj_bj_Jj¯≤bj¯+bj_bj_ηj¯+L2≤r0,WWWWWWWWWWWWWWWWWj=1,2,…,m,sup⁡t∈TxφiΔt=sup⁡t∈T∑j=1mpjitfjφn+jt−γjit+IitWWWW−ait∫−∞te−ait,σsWWWWWWWW·∑j=1mpjisfjWWWWWWWWWWW·φn+js−γjisWWWWWWWWWWWW+Iis∑j=1mpjiΔs∑j=1mpjitfjφn+jt−γjit+Iit≤sup⁡t∈T∑j=1mpji¯fj0+αjφn+jt−γjit+IitWWWW+ai¯∫−∞te−ait,σsWWWWWWWW·∑j=1mpji¯φn+js−γjisfj0WWWWWWWWWWWWW+αjφn+js−γjisWWWWWWWWWW+Iis∑j=1mpji¯Δs∑j=1mpji¯≤∑j=1mpji¯fj0+αjr0+Ii¯W+ai¯∑j=1mPji¯fj0+αjr0∫−∞te−ai_t,σsWWWWW·∑j=1mpji¯fj0+αjr0Δs+Ii¯ai_≤ai¯+ai_ai_ηi+max⁡1≤i≤nai¯+ai_ai_Ii¯≤ai¯+ai_ai_ηi+L1≤r0,WWWWWWWWW0WWWWWWi=1,2,…,n,sup⁡t∈Tyφn+jΔt=sup⁡t∈T∑i=1nqijtgiφit−ρijt+JjtWWWW−bjt∫−∞te−bjt,σsWWWWWWW0W·∑i=1nqijsgiWWWWWWWWWW·φis−ρijs+Jjs∑i=1nqijΔs∑i=1nqijtgiφit−ρijt+Jjt≤sup⁡t∈T∑i=1nqij¯gi0+βiφit−ρijt+Jjt+bj¯WWWW·∑i=1nqij¯∫−∞te−bjt,σsWWWWWWW·∑i=1nqij¯gi0+βiφis−ρijsWWWWWWWWW+Jjs∑i=1nqij¯Δs∫−∞te−bj≤∑i=1nqij¯gi0+βir0+Jj¯W+bj¯Jj¯bj_∫−∞te−bj_t,σsWWWWW·∑i=1nqij¯gi0+βir0Δs+Jj¯bj_≤bj¯+bj_bj_ηj¯+max⁡1≤j≤mbj¯+bj_bj_Jj¯≤bj¯+bj_bj_ηj¯+L2≤r0,WWWWWWWWWWWWWWWWj=1,2,…,m;

then, it follows from (37) that

(38) ΦφB=max⁡1≤i≤n, 1≤j≤msup⁡t∈Txφit,sup⁡t∈Tyφn+jt,WWWWWWWWWWsup⁡t∈TxφiΔt,sup⁡t∈Tyφn+jΔt≤r0.

Therefore, Φ(*E*) ⊂ *E*.

Taking *φ*, *ψ* ∈ *E* and combining conditions (*H*_1_) and (*H*_4_), we obtain that

(39) sup⁡t∈Txφit−xψit=sup⁡t∈T∑j=1mpji∫−∞te−ait,σsWWWWW·∑j=1mpjisWWWWWWWW·ψn+js−γjisfjφn+js−γjisWWWWWW0WW−fjψn+js−γjis∑j=1mpjiΔs≤sup⁡t∈T∑j=1mpji∫−∞te−ait,σsWWWWW·∑j=1mpjisαjφn+js−γjiswwwwwwwwwwwwwww−ψn+js−γjis∑j=1mpjiΔs≤sup⁡t∈T∫−∞te−ai_t,σs∑j=1mpji¯αjΔsφ−ψB≤Πiai_φ−ψB≤ai¯+ai_ai_Πiφ−ψB<φ−ψB,WWWWWWWWWWWWWWWWWi=1,2,…,n,sup⁡t∈Tyφn+jt−yψn+jt=sup⁡t∈T∫−∞te−bjt,σsWWWWW·∑i=1nqijsgiφis−ρijsWWWWWWWWWWW−giψis−ρijs∑i=1nqijΔs≤sup⁡t∈T∑i=1nqij∫−∞te−bjt,σsWWWWW·∑i=1nqijsβiφis−ρijswwwwwwwwwwwwwww−ψis−ρijs∑i=1nqijΔs≤sup⁡t∈T∫−∞te−bj_t,σs∑i=1nqij¯βiΔsφ−ψB≤Πj¯bj_φ−ψB≤bj¯+bj_bj_Πj¯φ−ψB<φ−ψB, j=1,2,…,m,sup⁡t∈Txφit−xψitΔ=sup⁡t∈TxφiΔt−xψiΔt≤sup⁡t∈T∑j=1mpjitfjφn+jt−γjitWWWWWWWW−fjψn+jt−γjitWWW+ai¯∑j=1mpjis∫−∞te−ai_t,σsWWWWWWW·∑j=1mpjisWWWWWWWWWW·fjφn+js−γjisWWWWWWWWWWW−fjs−γjisψn+jWWWWWWWWWWWWW·s−γjis∑j=1mpjisΔs≤sup⁡t∈T∑j=1mpjitαjWWWW·φn+jt−γjit−ψn+jt−γjitWWWW+ai¯∑j=1mpjis∫−∞te−ai_t,σsWWWWWWWW·∑j=1mpjisαjWWWWWWWWWWW·φn+js−γjisWWWWWWWWWWWW−ψn+js−γjis∑j=1mpjisΔs≤∑j=1mpji¯αjai¯+ai_ai_φ−ψB≤Πiai¯+ai_ai_φ−ψB<φ−ψB, i=1,2,…,n,sup⁡t∈Tyφn+jt−yψn+jtΔ=sup⁡t∈Tyφn+jΔt−yψn+jΔt≤sup⁡t∈T∑i=1nqijtgiφit−ρijt−giψit−ρijtWWWW+bj¯−giψis−ρijs∑i=1nqijs∫−∞te−bj_t,σsWWWWWWWW·∑i=1nqijs0000000000000000000000·giφis−ρijsWWWWWWWWW00000−giψis−ρijs∑i=1nqijsΔs≤sup⁡t∈T∑i=1nqijtβiφit−ρijt−ψit−ρijtWWW+bj¯∑i=1nqijs∫−∞te−bj_t,σsWWWWW·∑i=1nqijsβiWWWWWWWW·φis−ρijsWWWWWWWWW−ψis−ρijs∑i=1nqijsΔs≤∑i=1nqij¯βibj¯+bj_bj_φ−ψB≤Πj¯bj¯+bj_bj_φ−ψB<φ−ψB,WWWWWWWWWWWWWWWWWj=1,2,…,m.

Similarly, from (39) it follows that

(40) Φφ−ΦψBW=max⁡1≤i≤n,1≤j≤msup⁡t∈Txφit−xψit1,WWWWWW0Wsup⁡t∈Tyφn+jt−yψn+jt1W<φ−ψB.

By (40), we obtain that Φ is a contraction mapping from *E* to *E*. Since *E* is a closed subset of *B*, Φ has a fixed point in *E*, which means that (32) has a unique *C* ^1^-almost periodic solution in *E*. Then system (1) has a unique *C* ^1^-almost periodic solution in the region

(41) $$\begin{array}{c} E=\left\{\varphi \in B:{\left\|\varphi \right\|}_{B}\leq r_{0}\right\}. \end{array}$$

This completes the proof.

## 4. Exponential Stability of the *C* ^1^-Almost Periodic Solution

**Definition 16 .**

The *C* ^1^-almost periodic solution *z* ^*^(*t*) = (*x*_1_^*^(*t*), *x*_2_^*^(*t*),…, *x*_n_^*^(*t*), *y*_1_^*^(*t*),…, *y*_m_^*^(*t*))^*T*^ of system (1) with initial value *φ* ^*^(*t*) = (*φ*_1_^*^(*t*), *φ*_2_^*^(*t*),…, *φ*_n_^*^(*t*), *φ*_*n*+1_^*^(*t*),…, *φ*_*n*+m_^*^(*t*))^*T*^ is said to be globally exponentially stable. There exist a positive constant *λ* with ⊖*λ* ∈ *R* ^+^ and *M* > 1 such that every solution

(42) $$\begin{array}{c} z\left(t\right)={\left(x_{1}\left(t\right),x_{2}\left(t\right),\ldots ,x_{n}\left(t\right),y_{1}\left(t\right),\ldots ,y_{m}\left(t\right)\right)}^{T} \end{array}$$

of system (1) with any initial value

(43) φt=φ1t,φ2t,…,φnt,φn+1t,…,φn+mtT

satisfies

(44) $$\begin{array}{c} \left\|z\left(t\right)-z^{*}\left(t\right)\right\|\leq Me_{⊖\lambda }\left(t,t_{0}\right)\left\|\psi \right\|,\;\forall t\in {\left(0,+\infty \right)}_{T}, \end{array}$$

where

(45) ψsup⁡t∈−v,0Tmax⁡1≤i≤n+mφit−φi∗t,t0=max⁡−v,0T.

**Theorem 17 .**

Suppose that (*H*_1_)–(*H*_4_) hold and sup⁡_*t*∈*T*_ *μ*(*t*) < +*∞*; then, system (1) has a unique *C* ^1^-almost periodic solution which is globally exponentially stable.

**Proof**

According to Theorem 15, we know that (1) has a *C* ^1^-almost periodic solution

(46) z∗t=x1∗t,x2∗t,…,xn∗t,y1∗t,…,ym∗tT

with initial value *φ* ^*^(*t*) = (*φ*_1_^*^(*t*), *φ*_2_^*^(*t*),…, *φ*_n_^*^(*t*), *φ*_*n*+1_^*^(*t*),…, *φ*_*n*+m_^*^(*t*))^*T*^. Suppose that

(47) $$\begin{array}{c} z\left(t\right)={\left(x_{1}\left(t\right),x_{2}\left(t\right),\ldots ,x_{n}\left(t\right),y_{1}\left(t\right),\ldots ,y_{m}\left(t\right)\right)}^{T} \end{array}$$

is an arbitrary solution of (1) with initial value

(48) φt=φ1t,φ2t,…,φnt,φn+1t,…,φn+mtT.

Then it follows from system (1) that

(49) uiΔs+aisuisW=∑j=1mpjisfjvjs−γjis+yj∗s−γjisWWWW00WW−fjyj∗s−γjis,

(50) vjΔs+bjsvjsW=∑i=1nqijsgiuis−ρijs+xi∗s−ρijsWWW0WWW−gixi∗s−ρijs,

where *u*_*i*_(*s*) = *x*_*i*_(*s*) − *x*_i_^*^(*s*), *v*_*j*_(*s*) = *y*_*j*_(*s*) − *y*_j_^*^(*s*) and *i* = 1,2,…, *n*, *j* = 1,2,…, *m*, and the initial conditions of (49) and (50) are

(51) WWWWψis=φis−φi∗s,WWWWψn+js=φn+js−φn+j∗s,s∈−v,0T, i=1,2,…,n, j=1,2,…,m.

Let *H*_*i*_ and $\bar{H_{j}}$ be defined by

(52) HiϵW=ai_−ϵ−∑j=1mpji¯αjexp⁡ϵγ+sup⁡s∈Tμs,WWWWMWWi=1,2,…,n, ϵ∈0,+∞,Hj¯ϵW=bj_−ϵ−∑i=1nqij¯βiexp⁡ϵρ+sup⁡s∈Tμs,WWW00 WWj=1,2,…,m, ϵ∈0,+∞.

By (*H*_4_), we get

(53) Hi0=ai_−∑j=1mpji¯αj=ai_−Πi>0,WWWWWWW0Wi=1,2,…,n,Hj¯0=bj_−∑i=1nqij¯βi=bj_−Πj¯>0,WWWWWWW0Wj=1,2,…,m.

Since $H_{i},\bar{H_{j}}$ are continuous on [0, +*∞*) and *H*_*i*_(*ϵ*) → −*∞*, $\bar{H_{j}}\left(\epsilon \right)\to -\infty$ as *ϵ* → +*∞*, there exist $\epsilon_{i},\bar{\epsilon_{j}}>0$ such that *H*_*i*_(*ϵ*_*i*_) = 0, $\bar{H_{j}}\left(\bar{\epsilon_{j}}\right)=0$, and *H*_*i*_(*ϵ*) > 0 for *ϵ* ∈ (0, *ϵ*_*i*_) and $\bar{H_{j}}\left(\epsilon \right)>0$ for $\epsilon \in \left(0,\bar{\epsilon_{j}}\right)$. By choosing $\varepsilon =\min\left\{\epsilon_{1},\epsilon_{2},\ldots ,\epsilon_{n},\bar{\epsilon_{1}},\ldots ,\bar{\epsilon_{m}}\right\}$, we have

(54) WWWWHiε≥0,WWWWHj¯ε≥0,i=1,2,…,n, j=1,2,…,m.

So, we can choose a positive constant $0<\lambda <\min\left\{\varepsilon ,\min_{1\leq i\leq n}\left\{\underline{a_{i}}\right\},\min_{1\leq j\leq m}\left\{\underline{b_{j}}\right\}\right\}$ such that

(55) WWWWHiλ>0,WWWWHj¯λ>0,i=1,2,…,n, j=1,2,…,m,

which imply that

(56) 1ai_−λ∑j=1mpji¯αjexp⁡λγ+sup⁡s∈Tμs<1,WWWWWWWWWW0WWW i=1,2,…,n,1bj_−λ∑i=1nqij¯βiexp⁡λρ+sup⁡s∈Tμs<1,WWWWWWWW00WWWWj=1,2,…,m.

Multiplying (49) by *e*_−*a*_*i*__(*t*_0_, *σ*(*s*)) and integrating on [*t*_0_, *t*]_*T*_, by Lemma 12, we get

(57) uit=uit0e−ait,t0W+∫t0te−ait,σsWWW·∑j=1mpjisfjvjs−γjis+yj∗s−γjisWWWWWWWWW−fjyj∗s−γjis∑j=1mpjiΔs,WWWWWWWWWWWWWWW0WWi=1,2,…,n.

Similarly, multiplying (50) by *e*_−*b*_*j*__(*t*_0_, *σ*(*s*)) and integrating on [*t*_0_, *t*]_*T*_, we have

(58) vjt=vjt0e−bjt,t0W+∫t0te−bjt,σsWWW·∑i=1nqijsgiuis−ρijsWWWWWWWWWW+xi∗s−ρijsWWWWWWWW−gixi∗s−ρijs∑i=1nqijΔs,WWWWWWWWWWWWWMj=1,2,…,m.

Take $M>\max_{1\leq i\leq n,1\leq j\leq m}\left\{\underline{a_{i}}/\sum_{j=1}^{m}\bar{p_{ji}}\alpha_{j},\underline{b_{j}}/\sum_{i=1}^{n}\bar{q_{ij}}\beta_{i}\right\}$; then by (*H*_4_) we have *M* > 1. Thus, there exists $0<\lambda_{0}<\min\left\{\epsilon_{1},\epsilon_{2},\ldots ,\epsilon_{n},\bar{\epsilon_{1}},\ldots ,\bar{\epsilon_{m}}\right\}$ such that, for 0 < *λ* ≤ *λ*_0_,

(59) 1M−1ai_−λ∑j=1mpji¯αjexp⁡λγ+sup⁡s∈Tμs<0,WWWWWWWWWWWWWWWWi=1,2,…,n,1M−1bj_−λ∑i=1nqij¯βiexp⁡λρ+sup⁡s∈Tμs<0,WWWWWWWWWWWWWWWj=1,2,…,m.

It is easy to see that ⊖*λ* ∈ *R* ^+^ and

(60) uit=ψit≤ψ≤Me⊖λt,t0ψ,WWWWW  t∈−v,0T, i=1,2,…,n,vjt=ψn+jt≤ψ≤Me⊖λt,t0ψ,WWWW0W  t∈−v,0T, j=1,2,…,m,

which imply that

(61) zt−z∗t=max⁡1≤i≤n,1≤j≤m⁡uit,vjt≤Me⊖λt,t0ψ, t∈−v,0T.

Next, we claim that

(62) $$\begin{array}{c} \left\|z\left(t\right)-z^{*}\left(t\right)\right\|\leq Me_{⊖\lambda }\left(t,t_{0}\right)\left\|\psi \right\|,\;\forall t\in {\left(0,+\infty \right)}_{T}. \end{array}$$

If (62) is not true, then there must be some *t*_1_ ∈ (0, +*∞*)_*T*_, *p* > 1 and some *k* such that

(63) $$\begin{array}{c} \left\|z\left(t_{1}\right)-z^{*}\left(t_{1}\right)\right\|=\left|z_{k}\left(t_{1}\right)-z_{k}^{*}\left(t_{1}\right)\right|=pMe_{⊖\lambda }\left(t_{1},t_{0}\right)\left\|\psi \right\|, \end{array}$$

(64) zt−z∗t≤pMe⊖λt,t0ψ,WWWWWWWWW∀t∈−v,t1T.

By (57)–(64) and (*H*_2_)–(*H*_4_), we obtain

(65) uit1≤e−ait1,t0ψW+∫t0t1pMψe−ait1,σsWWW·∑j=1mpji¯αje⊖λs−γjis,t0Δs≤pMe⊖λt1,t0ψM·∑j=1mpji¯αje⊖λs−γ,σs1pMe−ait1,t0e⊖λt0,t1MMM+∫t0t1e−ait1,σseλt1,σsMMM·∑j=1mpji¯αje⊖λs−γ,σsΔs<pMe⊖λt1,t0ψM·∑j=1mpji¯1Me−ai⊕λt1,t0MMM+∑j=1mpji¯αjexp⁡⁡λγ+sup⁡s∈TμsMMM·∫t0t1e−ai⊕λt1,σsΔs∑j=1mpji¯≤pMe⊖λt1,t0ψM·∑j=1mpji¯1Me−ai⊕λt1,t0MMM+∑j=1mpji¯αjexp⁡λγ+sup⁡s∈TμsMMM·1−e−ai⊕λt1,t0ai_−λ∑j=1mpji¯≤pMe⊖λt1,t0ψM·1M−1ai_−λ∑j=1mpji¯αjexp⁡λγ+sup⁡s∈TμsMMM·e−ai⊕λt1,t0+1ai_−λMMM·∑j=1mpji¯αjexp⁡λγ+sup⁡s∈Tμs<pMe⊖λt1,t0ψ,vjt1≤e−bjt1,t0ψW+∫t0t1pMψe−bjt1,σsWWWWW·∑i=1nqij¯βie⊖λs−ρijs,t0Δs≤pMe⊖λt1,t0ψW·∑i=1nqij¯βie⊖λs−ρ,σs1pMe−bjt1,t0e⊖λt0,t1WW+∫t0t1e−bjt1,σseλt1,σsWW·∑i=1nqij¯βie⊖λs−ρ,σsΔs<pMe⊖λt1,t0ψW·∑i=1nqij¯βiexp⁡λρ+sup⁡s∈Tμs1Me−bj⊕λt1,t0WW+∑i=1nqij¯βiexp⁡λρ+sup⁡s∈TμsWW·∫t0t1e−bj⊕λt1,σsΔs∑i=1nqij¯βiexp⁡λρ+sup⁡s∈Tμs≤pMe⊖λt1,t0ψW·∑i=1nqij¯βiexp⁡λρ+sup⁡s∈Tμs1Me−bj⊕λt1,t0WW00+∑i=1nqij¯βiexp⁡λρ+sup⁡s∈TμsWWW·1−e−bj⊕λt1,t0bj_−λ∑i=1nqij¯βiexp⁡λρ+sup⁡s∈Tμs≤pMe⊖λt1,t0ψW·1M−1bj_−λ∑i=1nqij¯βiexp⁡λρ+sup⁡s∈TμsWWW·e−bj⊕λt1,t0+1bj_−λWWW·∑i=1nqij¯βiexp⁡λρ+sup⁡s∈Tμs<pMe⊖λt1,t0ψ.

Equations in (65) imply that

(66) zkt1−zk∗t1<pMe⊖λt1,t0ψ,WWWWWWW∀k∈1,2,…,n+m,

which contradicts (63), and so (62) holds. Hence, the *C* ^1^-almost periodic solution of system (1) is globally exponentially stable. Global exponential stability implies that the *C* ^1^-almost periodic solution is unique.

**Remark 18 .**

In [17, 25, 26, 29], the existence and stability of almost periodic solutions are studied for several classes of neural networks on almost periodic time scales. However, the almost periodic time scales used in [17, 25, 26, 29] are a kind of periodic time scales. So, the methods and the results of this paper are essentially new.

## 5. Some Examples

Consider the following neural network:

(67) xiΔt=−aitxit+∑j=12pjitfjyjt−γjit+Iit, t∈T,  i=1,2,yjΔt=−bjtyjt +∑i=12qijtgixit−ρijt+Jjt,ddddddddddddddddddt∈T,  j=1,2,

where

(68) f1xcos⁡3x+518,f2x=cos⁡3x+312,g1x=2−sin4x16,g2x=3−sin6x24.

**Example 1 .**

In (67), take *T* = *R*:

(69) a1t11+cos⁡⁡2t,a2t12−sint,b1t9−cos⁡t,b2t8+sint2,I1t2J1t=cos⁡t+3sint8,I2t4J2t=sin2t+cos⁡2t4,pjit2×217cos⁡t114sint114cos⁡t128sint,qijt2×216sint112cos⁡t112sint124sint.

Let *γ*_*ji*_, *ρ*_*ij*_  (*i*, *j* = 1,2) : *R* → *R* be arbitrary almost periodic functions; then, (*H*_2_)-(*H*_3_) hold. Let *α*_1_ = *α*_2_ = *β*_1_ = *β*_2_ = 1/4; then, (*H*_1_) holds. Next, let us check (*H*_4_); if we take *r*_0_ = 1, then

(70) max⁡a1¯+a1_a1_η1,a2¯+a2_a2_η2,b1¯+b1_b1_η1¯,b2¯+b2_b2_η2¯WW+max⁡L1,L2=2388+2344≈0.784<1=r0,0<Π1=356<1123=a1_a1¯+a1_<11=a1_,0<Π2=3112<1123=a2_a2¯+a2_<11=a2_,0<Π1¯=116<817=b1_b1¯+b1_<8=b1_,0<Π2¯=132<817=b2_b2¯+b2_<8=b2_.

**Figure 1:**
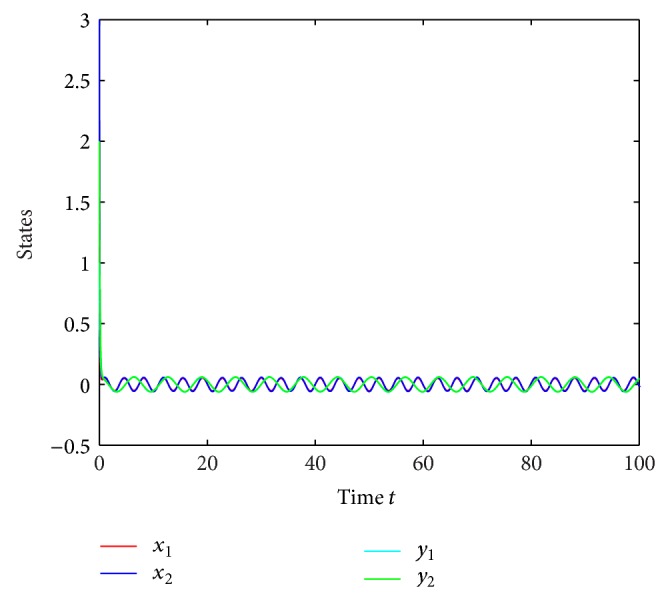
Responses of *x*_1_, *x*_2_, *y*_1_, *y*_2_ with continuous time *t*.

**Figure 2:**
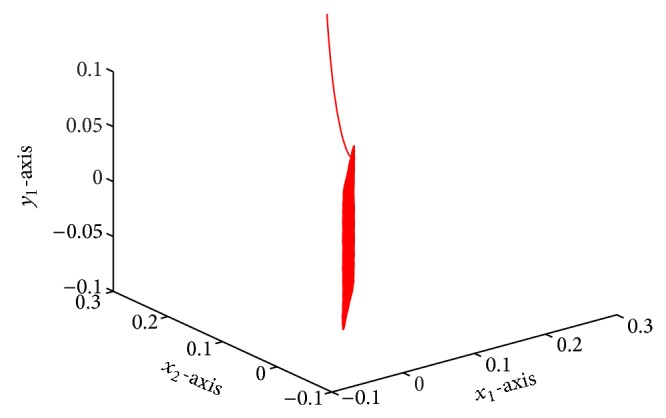
Responses of *x*_1_, *x*_2_, *y*_1_.

**Figure 3:**
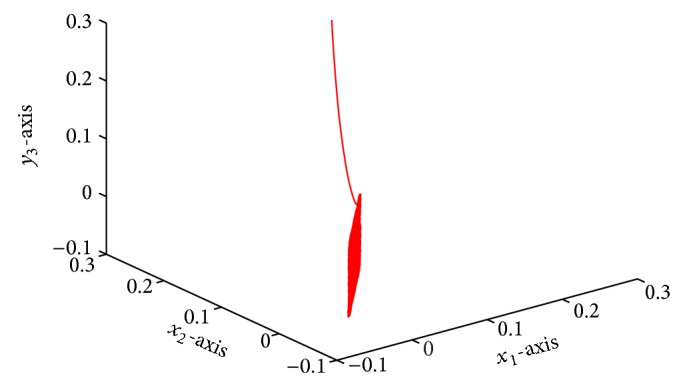
Responses of *x*_1_, *x*_2_, *y*_2_ with time *t*.

**Figure 4:**
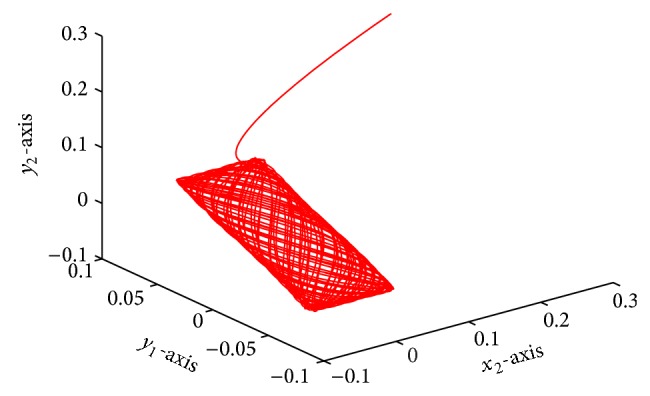
Responses of *x*_2_, *y*_1_, *y*_2_.

Thus, (*H*_4_) holds for *r*_0_ = 1. By Theorems 15 and 17, system (67) has a unique *C* ^1^-almost periodic solution in the region

(71) $$\begin{array}{c} E=\left\{\varphi \in B:{\left\|\varphi \right\|}_{B}\leq 1\right\}, \end{array}$$

which is globally exponentially stable (see Figures 1–4).

**Example 2 .**

In (67), take *T* = *Z*:

(72) a1t0.9−0.1sin3t,a2t0.8+0.1cos⁡2t,b1t0.6−0.1sint,b2t0.5+0.1cos⁡t4,I1tJ1t=sint+3cos⁡t16,I2t2J2t=2sint+2cos⁡t32,pjit2×217sint17sin2t128cos⁡t114sin⁡2t,qijt2×218sint124cos⁡2t148sint116cos⁡t.

Let *γ*_*ji*_, *ρ*_*ij*_  (*i*, *j* = 1,2) : *Z* → *Z* be arbitrary almost periodic functions; then, (*H*_2_)-(*H*_3_) hold. Let *α*_1_ = *α*_2_ = *β*_1_ = *β*_2_ = 1/4; then, (*H*_1_) holds. Next, let us check (*H*_4_); if we take *r*_0_ = 1, then

(73) max⁡a1¯+a1_a1_η1,a2¯+a2_a2_η2,b1¯+b1_b1_η1¯,b2¯+b2_b2_η2¯WW+max⁡L1,L2=2311920+1140≈0.395<1=r0,0<Π1=5112<817=a1_a1¯+a1_<0.8=a1_,0<Π2=356<817=a2_a2¯+a2_<0.8=a2_,0<Π1¯=7192<511=b1_b1¯+b1_<0.5=b1_,0<Π2¯=5192<511=b2_b2¯+b2_<0.5=b2_.

**Figure 5:**
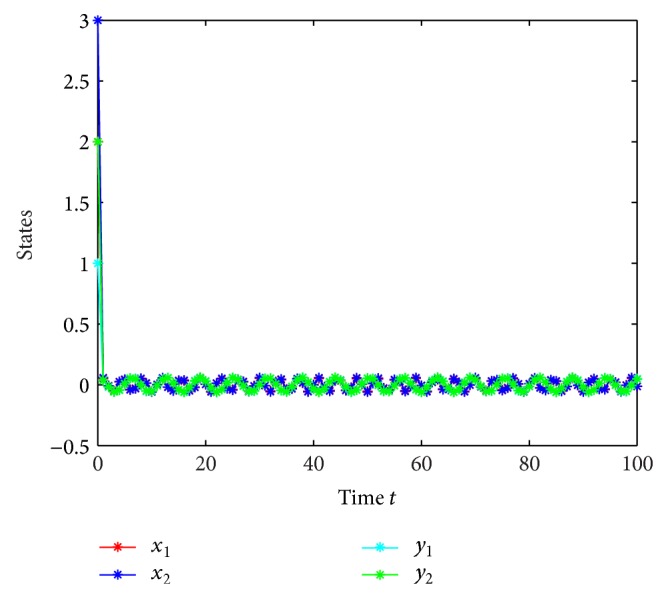
Responses of *x*_1_, *x*_2_, *y*_1_, *y*_2_ with discrete time *t*.

**Figure 6:**
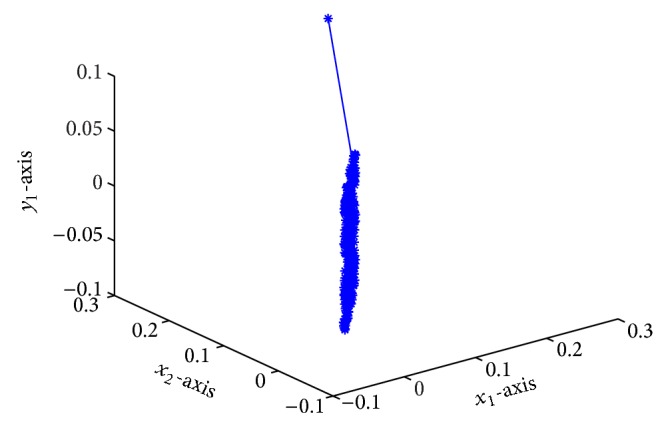
Responses of *x*_1_, *x*_2_, *y*_1_.

**Figure 7:**
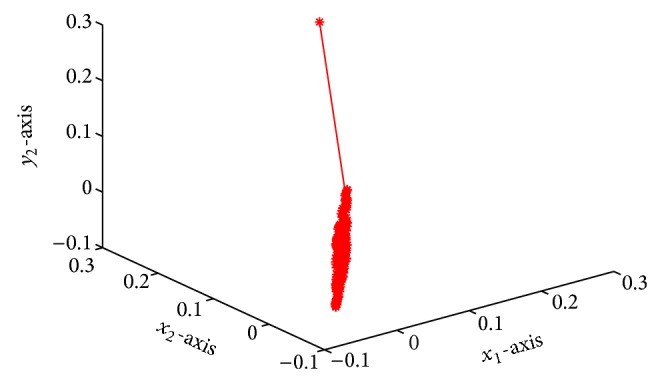
Responses of *x*_1_, *x*_2_, *y*_2_ with time *t*.

**Figure 8:**
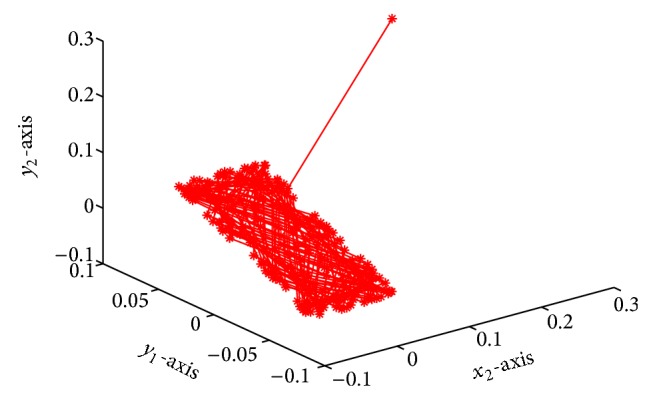
Responses of *x*_2_, *y*_1_, *y*_2_.

Thus, (*H*_4_) holds for *r*_0_ = 1. By Theorems 15 and 17, system (67) has a unique *C* ^1^-almost periodic solution in the region

(74) $$\begin{array}{c} E=\left\{\varphi \in B:{\left\|\varphi \right\|}_{B}\leq 1\right\}, \end{array}$$

which is globally exponentially stable (see Figures 5–8).

## 6. Conclusion

In this paper, by using calculus theory on time scales, a fixed point theorem, and differential inequality techniques, some sufficient conditions ensuring the existence and global exponential stability of *C* ^1^-almost periodic solutions for a class of neural networks with time-varying delays on a new type of almost periodic time scales are established. To the best of our knowledge, this is the first time to study the existence of *C* ^1^-almost periodic solutions of BAM neural networks on time scales. Our methods that are used in this paper can be used to study other types of neural networks, such as Cohen-Grossberg neural networks and fuzzy cellular neural networks.
